# Supplementary material for: Influenza A Virus Infection Reactivates Human Endogenous Retroviruses Associated with Modulation of Antiviral Immunity
Source: Viruses. 2022 Jul 21;14(7):1591. doi: 10.3390/v14071591 (PMC9320126; doi:10.3390/v14071591)
Supplement: Supplementary file 1 [file viruses-14-01591-s001.zip › MotifAnalysis.zip!/MotifOutput_SC35MdNS1/homerResults/motif10.similar.html]

motif10

## Information for motif10

A
G
T
C
A
C
G
T
C
G
T
A
A
C
T
G
A
G
T
C
A
C
G
T
A
C
T
G
A
G
T
C
  
Reverse Opposite:  

A
C
T
G
A
G
T
C
C
G
T
A
A
C
T
G
A
G
T
C
A
C
G
T
C
G
T
A
A
C
T
G
  

|  |  |
| --- | --- |
| p-value: | 1e-30 |
| log p-value: | -6.942e+01 |
| Information Content per bp: | 1.530 |
| Number of Target Sequences with motif | 23.0 |
| Percentage of Target Sequences with motif | 29.87% |
| Number of Background Sequences with motif | 322.9 |
| Percentage of Background Sequences with motif | 0.72% |
| Average Position of motif in Targets | 96.6 +/- 46.2bp |
| Average Position of motif in Background | 99.7 +/- 88.2bp |
| Strand Bias (log2 ratio + to - strand density) | 10.0 |
| Multiplicity (# of sites on avg that occur together) | 1.17 |
| Motif File: | file (matrix) reverse opposite |

### Similar de novo motifs found

|  |  |  |  |  |  |  |  |
| --- | --- | --- | --- | --- | --- | --- | --- |
| Rank | Match Score | Redundant Motif | P-value | log P-value | % of Targets | % of Background | Motif file |
| 1 | 0.614 | C T A G C G T A C T A G A T G C G T A C A G T C C G T A A C T G A G T C A C G T C T A G A C T G | 1e-12 | -27.874533 | 11.69% | 0.26% | motif file (matrix) |
| 2 | 0.637 | A C T G A G T C C G T A A C T G A G T C A C G T A C G T A T G C C G T A A G T C | 1e-5 | -13.583406 | 5.19% | 0.10% | motif file (matrix) |
